# Smart bio-implants for early detection and proactive management of cardiac Behçet’s disease

**DOI:** 10.1097/MS9.0000000000004469

**Published:** 2025-12-04

**Authors:** Hamdia Gul Aslam, Hamza Sajid, Noor Un Nisa Irshad, Sakan Binte Imran

**Affiliations:** a Department of Medicine, Allama Iqbal Medical College, Lahore, Pakistan; b Department of Medicine, Jinnah Sindh Medical University, Karachi, Pakistan; c Department of Medicine, Sir Salimullah Medical College, Dhaka, Bangladesh

*To the Editor,*

Behcet’s disease (BD) is an autoinflammatory disorder characterized by widespread vasculitis. Its manifestations include recurrent oral and genital ulcers, usually accompanied by ocular symptoms such as uveitis. Other than these features, BD can affect nearly any organ system, which includes musculoskeletal involvement like arthritis, neurological complications spanning from headaches to meningoencephalitis, and cardiac lesions such as pericarditis and myocarditis. Patients may also experience pulmonary issues like pulmonary artery aneurysms and gastrointestinal system involvement, which presents with inflammatory ulcers similar to Crohn’s disease^[1]^. Cardiac involvement in BD is also referred to as cardiac BD. Cardiac involvement may occur in the form of endocarditis, myocarditis, pericarditis, endomyocardial fibrosis, coronary arteritis, and valvular disease^[2]^. Although cardiovascular involvement in BD is rare, it is related to mortality as high as 20% at 1 year after diagnosis. Cardiac BD, if undiagnosed, can cause serious and ultimately life-threatening conditions such as coronary artery aneurysms, myocardial infarction, and intracardiac thrombosis. Almost 40% of patients with cardiac BD develop significant structural heart disease^[3]^. The failure to diagnose this involvement can cause a potentially manageable condition to advance into a fatal condition. Early detection and proper management of cardiovascular involvement can enhance patient prognosis. It is thus crucial to diagnose cardiovascular involvement in BD as soon as possible.

The current diagnostic basis for cardiac involvement in BD includes advanced imaging modalities, primarily echocardiography and cardiac magnetic resonance (CMR). These techniques are considered the gold standards for visualizing structural anomalies, such as valvular lesions and coronary artery aneurysms, and for detecting tissue-level inflammation and edema through late gadolinium enhancement and T2-weighted sequences on CMR^[4]^. However, these modalities possess critical limitations in disease management. First, they are inconsistent; hence, they easily miss transient, yet damaging, flares of active myocarditis or pericarditis. As a result, diagnosis frequently occurs reactively, only taking place after significant clinical damage has built up, resulting in irreversible myocardial fibrosis or a severe event^[5]^. Second, the logistical and economic challenges of regular CMR and specialist echocardiographic studies render continuous monitoring impractical for most health care systems and patients. Furthermore, echocardiography is highly operator dependent and can lack the sensitivity to detect early tissue changes, while CMR is often contraindicated in specific patient groups and may only identify permanent, chronic damage rather than the acute inflammatory activity that precedes it^[6]^. This highlights the need to shift from the reactive, episodic screening of CMR and echocardiographic studies to continuous monitoring to prevent the progression of silent cardiac disease in BD.

To address this monitoring gap, the use of a new class of small, biocompatible, and wireless bio-implants within the pericardial space or on the myocardial surface is suggested. These smart implants use a multi-sensor approach to provide a comprehensive and real-time picture of cardiac inflammation. Bio-impedance sensors continuously track local tissue edema, a hallmark of active vasculitis, by detecting small changes in electrical conductivity. Simultaneously, electrochemical biosensors, having immobilized aptamers, can detect specific inflammatory biomarkers such as IL-6 and CRP directly at the source, offering a molecular-level diagnosis of disease activity. To create a comprehensive physiological picture, microelectromechanical systems (MEMS) -based physiologic sensors are used in conjunction with this to monitor data such as intracardiac pressure, contractile strain, and local temperature. Recent advancements have shown that it is possible to create flexible, minimally invasive sensors for ongoing cardiac monitoring^[7]^. The long-term feasibility of implanted hemodynamic sensors in the human heart is demonstrated by their clinical success^[8]^. However, the actual strength of this system lies in its integration into an electronic telehealth environment. In such a system, the patient’s smartphone reads data from the implant and sends it to their health care providers. It allows for early intervention before any symptoms even begin to appear by sending a notice when inflammation levels begin to increase. Over time, data accumulated allows for a clear, long-term profile of the disease, enabling treatment to be adapted to each person’s condition. It eventually alters how patients view their treatment by providing them with clear details concerning their health and motivating them to become active, cooperative participants in their own treatment^[9]^.

In conclusion, smart bio-implants offer not only data but also a chance to change the way of dealing with Behçet’s patients who are at cardiac risk. However, this vision can only be brought to reality by multi-disciplinary integration of rheumatology, cardiology, and bioengineering. This approach will turn the uncertainty of a complex autoinflammatory condition into a manageable disease in the future if applied correctly. This article aligns with the TITAN Guidelines on the need for transparency in AI use in health care^[10]^.

## Data Availability

Not applicable.
